# Association of Timing of Electrocardiogram Acquisition After Return of Spontaneous Circulation With Coronary Angiography Findings in Patients With Out-of-Hospital Cardiac Arrest

**DOI:** 10.1001/jamanetworkopen.2020.32875

**Published:** 2021-01-11

**Authors:** Enrico Baldi, Sebastian Schnaubelt, Maria Luce Caputo, Catherine Klersy, Christian Clodi, Jolie Bruno, Sara Compagnoni, Claudio Benvenuti, Hans Domanovits, Roman Burkart, Rosa Fracchia, Roberto Primi, Gerhard Ruzicka, Michael Holzer, Angelo Auricchio, Simone Savastano

**Affiliations:** 1 Department of Molecular Medicine, Section of Cardiology, University of Pavia, Pavia, Italy; 2 Cardiac Intensive Care Unit, Arrhythmia and Electrophysiology and Experimental Cardiology, Fondazione Istituto di Ricovero e Cura a Carattere Scientifico (IRCCS) Policlinico San Matteo, Pavia, Italy; 3 Department of Emergency Medicine, Medical University of Vienna, Vienna, Austria; 4 Division of Cardiology, Cardiocentro Ticino, Lugano, Switzerland; 5 Service of Clinical Epidemiology and Biometry, Fondazione IRCCS Policlinico San Matteo, Pavia, Italy; 6 Fondazione Ticino Cuore, Breganzona, Switzerland; 7 Division of Cardiology, Fondazione IRCCS Policlinico San Matteo, Pavia, Italy

## Abstract

**Importance:**

Electrocardiography (ECG) is an important tool to triage patients with out-of-hospital cardiac arrest (OHCA) after return of spontaneous circulation (ROSC). An immediate coronary angiography after ROSC is recommended only in patients with an ECG that is diagnostic of ST-segment elevation myocardial infarction (STEMI). To date, the benefit of this approach has not been demonstrated in patients with a post-ROSC ECG that is not diagnostic of STEMI.

**Objective:**

To assess whether the time from ROSC to ECG acquisition is associated with the diagnostic accuracy of ECG for STEMI.

**Design, Setting, and Participants:**

This retrospective, multicenter cohort study (the Post-ROSC Electrocardiogram After Cardiac Arrest study) analyzed consecutive patients older than 18 years who were resuscitated from OHCA between January 1, 2015, and December 31, 2018, and were admitted to 1 of the 3 participating centers in Europe (Pavia, Italy; Lugano, Switzerland; and Vienna, Austria).

**Exposure:**

Only patients who underwent coronary angiography during hospitalization and who acquired a post-ROSC ECG before the angiography were enrolled. Patients with a nonmedical cause of OHCAs were excluded.

**Main Outcomes and Measures:**

The primary end point was false-positive ECG findings, defined as the percentage of patients with post-ROSC ECG findings that met STEMI criteria but who did not show obstructive coronary artery disease on angiography that was worthy of percutaneous coronary angioplasty.

**Results:**

Of 586 consecutive patients who were admitted to the 3 participating centers, 370 were included in the analysis (287 men [77.6%]; median age, 62 years [interquartile range, 53-70 years]); 121 (32.7%) were enrolled in the participating center in Pavia, Italy; 38 (10.3%) in Lugano, Switzerland; and 211 (57.0%) in Vienna, Austria. The percentage of false-positive ECG findings in the first tertile of ROSC to ECG time (≤7 minutes) was significantly higher than that in the second (8-33 minutes) and third (>33 minutes) tertiles: 18.5% in the first tertile vs 7.2% in the second (odds ratio [OR], 0.34; 95% CI, 0.13-0.87; *P* = .02) and 5.8% in the third (OR, 0.27; 95% CI, 0.15-0.47; *P* < .001). These differences remained significant when adjusting for sex (≤7 minutes: reference; 8-33 minutes: OR, 0.32; 95% CI, 0.12-0.85; *P* = .02; >33 minutes: OR, 0.26; 95% CI, 0.14-0.47; *P* < .001), age (≤7 minutes: reference; 8-33 minutes: OR, 0.34; 95% CI, 0.13-0.89; *P* = .03; >33 minutes: OR, 0.27; 95% CI, 0.15-0.46; *P* < .001), number of segments with ST-elevation (≤7 minutes: reference; 8-33 minutes: OR, 0.35; 95% CI, 0.15-0.81; *P* = .01; >33 minutes: OR, 0.28; 95% CI, 0.15-0.52; *P* < .001), QRS duration (≤7 minutes: reference; 8-33 minutes: OR, 0.35; 95% CI, 0.14-0.87; *P* = .02; >33 minutes: OR, 0.27; 95% CI, 0.15-0.48; *P* < .001), heart rate (≤7 minutes: reference; 8-33 minutes: OR, 0.35; 95% CI, 0.13-0.93; *P* = .04; >33 minutes: OR, 0.29; 95% CI, 0.15-0.55; *P* < .001), epinephrine administered (≤7 minutes: reference; 8-33 minutes: OR, 0.35; 95% CI, 0.13-0.98; *P* = .045; >33 minutes: OR, 0.27; 95% CI, 0.16-0.48; *P* < .001), shockable initial rhythm (≤7 minutes: reference; 8-33 minutes: OR, 0.35; 95% CI, 0.13-0.96; *P* = .04; >33 minutes: OR, 0.26; 95% CI, 0.15-0.46; *P* < .001), and 3 or more shocks administered (≤7 minutes: reference; 8-33 minutes: OR, 0.36; 95% CI, 0.13-1.00; *P* = .05; >33 minutes: OR, 0.27; 95% CI, 0.16-0.48; *P* < .001) in bivariable analyses.

**Conclusions and Relevance:**

This study suggests that early ECG acquisition after ROSC in patients with OHCA is associated with a higher percentage of false-positive ECG findings for STEMI. It may be reasonable to delay post-ROSC ECG by at least 8 minutes after ROSC or repeat the acquisition if the first ECG is diagnostic of STEMI and is acquired early after ROSC.

## Key Points

**Question:**

Is the time from the return of spontaneous circulation (ROSC) to electrocardiogram (ECG) acquisition associated with the percentage of false-positive ECG findings for ST-segment elevation myocardial infarction (STEMI) in patients who experience out-of-hospital cardiac arrest?

**Findings:**

In this cohort study of 370 patients who were resuscitated from out-of-hospital cardiac arrest, the percentage of false-positive ECG findings among those performed 7 minutes or less after ROSC (18.5%) was significantly higher than those performed between 8 and 33 minutes (7.2%) and over 33 minutes (5.8%) after ROSC.

**Meaning:**

Results of this study suggest that early ECG acquisition after ROSC is associated with a higher percentage of false-positive ECG findings for STEMI after out-of-hospital cardiac arrest.

## Teaser

This cohort study evaluates the association between the time from the return of spontaneous circulation to electrocardiogram (ECG) acquisition and the diagnostic accuracy of ECG for ST-segment elevation myocardial infarction in patients who experience out-of-hospital cardiac arrest.

## Introduction

Out-of-hospital cardiac arrest (OHCA) is one of the leading causes of death worldwide, with an estimated annual incidence of OHCAs treated by emergency medical services (EMS) ranging from 30.0 to 97.1 individuals per 100 000 inhabitants.^1,2^ The cornerstone in the treatment of cardiac arrest is represented by the chain of survival.^3^ The chain of survival is the linkage of the actions to be implemented, from the community response to in-hospital treatment, to improve the chance of achieving the return of spontaneous circulation (ROSC) and consequently the chances of survival.^4,5^ Starting in 2010, the European Resuscitation Council and the American Heart Association have focused on post-ROSC treatment by adding specific chapters to their resuscitation guidelines, and the American Heart Association added a fifth link to the classic chain of survival.^6,7^ A series of diagnostic and therapeutic actions must be implemented in the post-ROSC phase both to stabilize the patient and to discover and treat the underlying disease that led to the cardiac arrest.^6,8^ In this context, electrocardiography (ECG) represents the first and easiest diagnostic tool, and it should be acquired immediately after achievement of ROSC to identify the need for an urgent coronary angiography, which is indicated only in the case of ST-segment elevation myocardial infarction (STEMI).^9^ However, neither the American nor the European resuscitation guidelines provide specific instructions about the best time for ECG acquisition after ROSC; they only recommend the recording of a 12-lead ECG as soon as possible after ROSC.^6,8,9^

Despite the absence of data in the literature, it is reasonable to assume that, in the early post-ROSC phase, the ECG could reflect the ischemia secondary to cardiac arrest more than or in addition to ischemia due to coronary artery pathology, possibly leading to overdiagnosis of STEMI (false-positive ECG findings). This possibility represents a important topic because, in many cases, the choice made to transfer patients to a hospital that offers percutaneous coronary intervention–mediated reperfusion therapy 24 hours per day for 7 days per week (hub center) or to a hospital with no percutaneous coronary intervention (spoke center) depends on the diagnosis of STEMI. Furthermore, coronary angiography requires the administration of drugs (ie, heparin sodium) that could be potentially harmful in other medical causes of OHCA (eg, brain hemorrhage and aortic dissection).

The primary aim of this cohort study was to assess the association of the time from ROSC to ECG acquisition with the percentage of false-positive ECG findings for STEMI. The secondary aim was to identify the best timing for post-ROSC ECG acquisition to better select patients who require urgent percutaneous coronary angioplasty (PTCA) and thereby to minimize rates of false-positive ECG findings and maximize rates of true-negative ECG findings.

## Methods

### Type of Study and Centers Selection

This retrospective, multicenter cohort study (the Post-ROSC Electrocardiogram After Cardiac Arrest [PEACE] study) was endorsed by the European Resuscitation Council Research Net and approved by the ethics committee of the Fondazione Istituto di Ricovero e Cura a Carattere Scientifico (IRCCS) Policlinico San Matteo and the other participating centers. Considering the retrospective nature of the study and that the patients were already enrolled in local registries, no additional informed consent was required. The study followed the Strengthening the Reporting of Observational Studies in Epidemiology (STROBE) reporting guideline.

We considered all consecutive patients older than 18 years who received resuscitation for OHCA between January 1, 2015, and December 31, 2018, and who were admitted to 1 of the 3 participating centers in Europe (Fondazione IRCCS Policlinico San Matteo in Pavia, Italy; Cardiocentro Ticino in Lugano, Switzerland; and Medical University of Vienna in Vienna, Austria). We defined OHCA as cessation of cardiac mechanical activity as confirmed by the absence of signs of circulation that occurs outside a hospital setting. Only patients who underwent coronary angiography during hospitalization and who had post-ROSC ECG performed before the angiography were enrolled in the study. Patients who experienced OHCA with a nonmedical cause according to the 2014 Utstein style^10^ were excluded.

### Data Collection

The variables associated with OHCA and the outcomes were collected according to the 2014 Utstein style recommendations.^10^

The prehospital data related to OHCA were obtained from the prospective registries of the Fondazione IRCCS Policlinico San Matteo (Cardiac Arrest Registry of the Lombardy Region [Lombardia CARe]) and the Cardiocentro Ticino (Ticino Registry Cardiac Arrest [TiReCa]). Data for the patients admitted to the Medical University of Vienna were retrieved from the medical records of that hospital.

Data from the first ECG acquired after ROSC were collected as were records of the coronary angiography performed during hospitalization. If the first ECG could not be evaluated because of artifacts or technical issues and was not recoverable, the second ECG was considered, as long as it was performed before the angiography. The ROSC to ECG time (expressed in minutes) was calculated as the time elapsed between the ROSC and the acquisition of the ECG.

### Evaluation of ECG Findings

Two cardiologists at each participating center evaluated all ECGs; these cardiologists were blinded to both the coronary angiography findings and the time elapsed between the ROSC and the ECG acquisition. In case of doubtful interpretation, a third cardiologist was asked to provide an additional interpretation. The rhythm, heart rate, QRS duration, corrected QT value, intraventricular conduction, arrhythmias, and segments with ST elevation (anterior, lateral, posterior, inferior, and right) were analyzed for each ECG. In addition, each ECG was categorized as either diagnostic or not diagnostic of STEMI according to the criteria of the electrocardiographic diagnosis of STEMI recommended by the 2017 European Society of Cardiology guidelines for the management of acute myocardial infarction.^9^ The isolated ST-segment depression in leads V1 to V3 was considered diagnostic of posterior STEMI without the need of confirmation by the posterior leads, considering the clinical picture of a resuscitated patients with OHCA. The criteria proposed by Sgarbossa et al^11^ were considered in the presence of a left bundle branch block.

### Evaluation of Coronary Angiography Findings

For patients who had undergone coronary angiography, we assessed the presence of significant coronary stenosis, the number of vessels in which stenosis was present, and the execution of any PTCA by analyzing the procedure report. If the report was not exhaustive, an interventional cardiologist who was blinded to the patient’s post-ROSC ECG was asked to evaluate the coronary angiography. Coronary artery stenosis was defined as significant if it was greater than 50% for the left main coronary artery and greater than or equal to 75% for the other coronary vessels.^12,13^

### Definitions

On the basis of the post-ROSC ECG and coronary angiography findings, the study population was divided into 4 groups. Those with true-positive ECG findings were patients who had post-ROSC ECG findings that met STEMI criteria and had obstructive coronary artery disease worthy of PTCA confirmed by angiography. Patients with true-negative ECG findings included those who had post-ROSC ECG findings that did not meet STEMI criteria and did not have obstructive coronary artery disease worthy of PTCA confirmed by angiography. Patients with false-positive ECG findings included those who had post-ROSC ECG findings that met STEMI criteria but did not have obstructive coronary artery disease worthy of PTCA by angiography. Patients with false-negative ECG findings included those who had post-ROSC ECG findings that did not meet STEMI criteria but who had obstructive coronary artery disease that was worthy of PTCA on angiography.

### Statistical Analysis

Study data were collected and managed after being anonymized using REDCap electronic data capture tools hosted at Fondazione IRCCS Policlinico San Matteo.^14,15^ The categorical variables were compared with the χ^2^ test and presented as a number (percentage). The continuous variables were tested for normal distribution with the D’Agostino-Pearson test. If normally distributed, they were compared with an unpaired, 2-tailed *t* test and presented as mean (SD); otherwise, they were compared with the Mann-Whitney test and presented as median (interquartile range [IQR]). Logistic regression was used to analyze the association between time from ROSC and false-positive ECG findings. We fitted a multivariable model using time from ROSC categorized into tertiles. We also fitted a second model with time on a continuous scale using fractional polynomial to graphically confirm the shape of the risk associated with time from ROSC. Given the low number of false-positive ECG findings, we could not use a multivariable analysis to control for confounding. Instead, we adjusted the association of time from ROSC to false-positive ECG findings for confounding in a series of bivariable models. The following were a priori–defined potential confounders: sex, age of 62 years or younger or older than 62 years, number of segments with ST elevation of 1 or fewer or more than 1, QRS duration of 120 milliseconds or less or more than 120 milliseconds, heart rate of 100 beats per minute or fewer or more than 100 beats per minute, amount of epinephrine administered of 1 mg or less or greater than 1 mg, initial rhythm and number of shocks administered of fewer than 3 or 3 or more. For all logistic models, Huber-White robust SEs were computed while clustering on center to account for within-center correlation of observations.

Statistical analyses were performed using MedCalc, version 19.2 (MedCalc Software Ltd), and Stata, version 16 (StataCorp LLC). All tests were 2-sided, and *P* < .05 was considered statistically significant.

## Results

**Figure 1.**
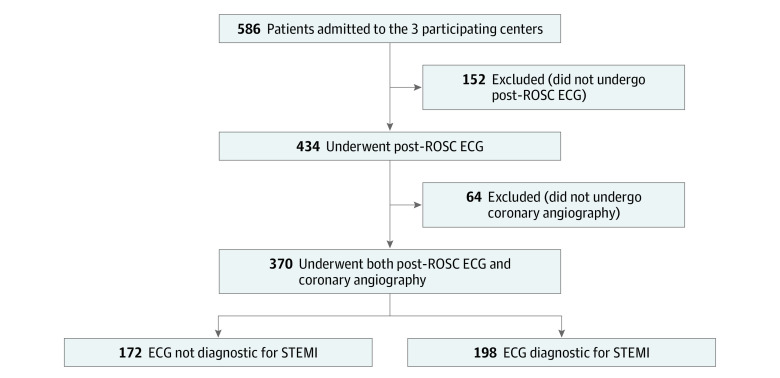
Flowchart of the Study Participants ECG indicates electrocardiography; ROSC, return of spontaneous circulation; STEMI, ST-segment elevation myocardial infarction.

Of the 586 consecutive patients who were admitted to the 3 participating centers, 152 (25.9%) did not undergo a post-ROSC ECG before coronary angiography and 64 (10.9%) did not receive coronary angiography and thus were excluded. As a result, the final study population was composed of 370 patients, in whom 172 ECGs were not diagnostic of STEMI and 198 ECGs were diagnostic of STEMI (Figure 1). Among the 370 patients, 121 (32.7%) were enrolled in Pavia, Italy; 38 (10.3%) in Lugano, Switzerland; and 211 (57.0%) in Vienna, Austria. Of these patients, 287 were men (77.6%) and 83 were women (22.4%), with a median age of 62 years (IQR, 53-70 years). The patient and OHCA characteristics of the final population are presented in Table 1. OHCA occurred more frequently at home (187 [50.6%]), was witnessed by bystanders (267 [72.2%]) or the EMS (50 [13.5%]), and had a shockable initial rhythm (312 [84.8%]). Cardiopulmonary resuscitation was initiated by a bystander in 233 cases (73.3%). The percentage of patients who survived to hospital discharge with favorable neurological outcome was 57.3% (n = 212). The patient and OHCA characteristics according to the different tertiles of time from ROSC to post-ROSC ECG are presented in eTable 1 in the Supplement.

**Table 1.** Characteristics of the Whole Study Population and Comparison by Post-ROSC ECG Findings That Are Diagnostic or Not Diagnostic of STEMI^a^

Variable	Whole population (N = 370)	Post-ROSC ECG		*P* value
		Diagnostic of STEMI (n = 198)	Not diagnostic of STEMI (n = 172)	
Health care center location				
Pavia, Italy	121 (32.7)	91 (46.0)	30 (17.4)	<.001
Lugano, Switzerland	38 (10.3)	22 (11.1)	16 (9.3)	
Vienna, Austria	211 (57.0)	85 (42.9)	126 (73.3)	
Male	287 (77.6)	158 (79.8)	129 (75.0)	.27
Age, median (IQR), y	62 (53-70)	62 (54-70)	60.5 (52-71)	.37
OHCA location				
Home	187 (50.6)	124 (62.6)	63 (36.6)	<.001
Public building	88 (23.8)	33 (16.7)	55 (32.0)	
Work or office	9 (2.4)	4 (2.0)	5 (2.9)	
Street	60 (16.2)	27 (13.6)	33 (19.2)	
Sport	3 (0.8)	1 (0.5)	2 (1.2)	
Others	16 (4.3)	6 (3.0)	10 (5.8)	
Unknown	7 (1.9)	3 (1.5)	4 (2.3)	
EMS arrival time, median (IQR), min	10 (8-12)	14 (9-42)	24 (10-51)	.20
OHCA witnessed				
No	53 (14.3)	24 (12.1)	29 (16.9)	.03
Yes, by bystander	267 (72.2)	139 (70.2)	128 (74.4)	
Yes, by EMS	50 (13.5)	35 (17.7)	15 (8.7)	
Bystander CPR^b^	233 (73.3)	113 (70.2)	120 (76.4)	.21
Shockable initial rhythm	312 (84.8)	174 (88.8)	138 (80.2)	.04
Epinephrine dose administered, median (IQR), mg	1 (0-3)	1 (0-3)	1 (0-2)	.01
Shocks administered, median (IQR), No.	2 (1-5)	3 (1-5)	2 (1-4)	.004
Survival at hospital discharge	244 (65.9)	126 (64.6)	118 (69.8)	.29
Survival at hospital discharge with good neurological outcome^c^	212 (57.3)	105 (53.0)	106 (61.6)	.09

Abbreviations: CPC, cerebral performance category; CPR, cardiopulmonary resuscitation; ECG, electrocardiography; EMS, emergency medical services; IQR, interquartile range; OHCA, out-of-hospital cardiac arrest; ROSC, return of spontaneous circulation; STEMI, ST-segment elevation myocardial infarction.

^a^ Data are presented as number (percentage) of individuals unless otherwise indicated.

^b^ Excluding EMS-witnessed.

^c^ CPC 1 for good cerebral performance, and CPC 2 for moderate cerebral disability.

### Post-ROSC ECG Diagnostic of STEMI

Dividing the population according to whether or not post-ROSC ECG was diagnostic of STEMI, we found that OHCA occurred more frequently at home (124 [62.6%] vs 63 [36.6%]; *P* < .001) and was more frequently witnessed by the EMS (35 [17.7%] vs 15 [8.7%]; *P* = .03) in the group with diagnostic ECGs than in the group without diagnostic ECGs. In the group with diagnostic ECGs vs the group without, the initial rhythm was more frequently shockable (174 [88.8%] vs 138 [80.2%]; *P* = .04), a higher median number of shocks were delivered (3 [IQR, 1-5] vs 2 [IQR, 1-4]; *P* = .004), and a higher median dose of epinephrine was administered (1 mg [IQR, 0-3 mg] vs 1 mg [IQR 0-2 mg]; *P* = .01). No differences were observed between the 2 groups regarding sex, age, EMS arrival time, bystander CPR rate, and outcome (Table 1).

The ECG and the coronary angiography characteristics are presented in Table 2. A PTCA was performed for 159 patients (80.3%) with a post-ROSC ECG diagnostic of STEMI and for 98 patients (57.0%) with a post-ROSC ECG not diagnostic of STEMI. Features of both ECG and the coronary angiography according to the different tertiles of time from ROSC to post-ROSC ECG are presented in eTable 2 in the Supplement.

**Table 2.** Electrocardiography and Coronary Angiography Findings^a^

Finding	Whole population (N = 370)	Post-ROSC ECG		*P* value
		Diagnostic of STEMI (n = 198)	Not diagnostic of STEMI (n = 172)	
**Post–ROSC ECG**				
ROSC to ECG time, median (IQR), min	15.5 (6-40)	8 (4-29)	31.5 (10-48.5)	<.001
Rhythm				
Sinus rhythm	269 (72.9)	139 (70.6)	130 (75.6)	.38
Atrial fibrillation or atrial tachycardia	80 (21.7)	47 (23.9)	33 (19.2)	
Junctional or ventricular rhythm	16 (4.3)	10 (5.1)	6 (3.5)	
Paced	4 (1.1)	1 (0.5)	3 (1.7)	
ECGs diagnostic of STEMI	198 (53.5)	NA	NA	NA
Heart rate, median (IQR), bpm	98 (78-115)	100 (79-120)	97 (78-112)	.23
QRS duration, median (IQR), ms	112 (96-140)	110 (90-140)	113.5 (100-140)	.38
QTc value, median (IQR), ms	462 (423-500)	450 (415-488)	472.5 (440.5-511.5)	<.001
Intraventricular conduction				
Normal	240 (64.9)	133 (67.2)	107 (62.2)	.19
Left bundle branch block	45 (12.2)	21 (10.6)	24 (13.9)	
Right bundle branch block	64 (17.3)	29 (14.6)	35 (20.3)	
Bifascicular block	8 (2.2)	5 (2.5)	3 (1.7)	
Others	13 (3.5)	10 (5.1)	3 (1.7)	
Arrhythmias^b^				
None	248 (92.5)	123 (89.1)	125 (96.2)	.03
Ventricular ectopy	14 (5.2)	12 (8.7)	2 (1.5)	
Supraventricular ectopy	6 (2.2)	3 (2.2)	3 (2.3)	
No. of segments with ST elevation				
None	147 (39.7)	1 (0.5)	146 (84.9)	<.001
1	104 (28.1)	81 (40.9)	23 (13.4)	
2	84 (22.7)	81 (40.9)	3 (1.7)	
3	28 (7.6)	28 (14.1)	0 (0)	
4	7 (1.9)	7 (3.5)	0 (0)	
**Coronary angiography**				
ECG angiography time, median (IQR), min	99 (64-206)	84 (58-115)	147 (78.5-752.5)	<.001
Normal coronary angiography	71 (19.2)	16 (8.1)	55 (32.0)	<.001
No. of vessels with significant stenosis				
None	80 (21.6)	18 (9.1)	62 (36.0)	<.001
1	113 (30.5)	79 (39.9)	34 (19.8)	
2	81 (21.9)	50 (25.3)	31 (18.0)	
3	96 (25.9)	51 (25.8)	45 (26.2)	
No. of vessels treated with PTCA				
None	113 (30.5)	39 (19.7)	74 (43)	<.001
1	153 (41.3)	114 (57.6)	39 (22.7)	
2	52 (14.1)	24 (12.1)	28 (16.3)	
3	52 (14.1)	21 (10.6)	31 (18.0)	

Abbreviations: ECG, electrocardiography; IQR, interquartile range; NA, not applicable; PTCA, percutaneous coronary angioplasty; QTc, corrected QT interval; ROSC, return of spontaneous circulation; STEMI, ST-segment elevation myocardial infarction.

^a^ Data are presented as number (percentage) of individuals unless otherwise indicated.

^b^ Considering only ECG with sinus rhythm.

### False-Positive ECG Findings and Time From ROSC to Post-ROSC ECG Acquisition

**Figure 2.**
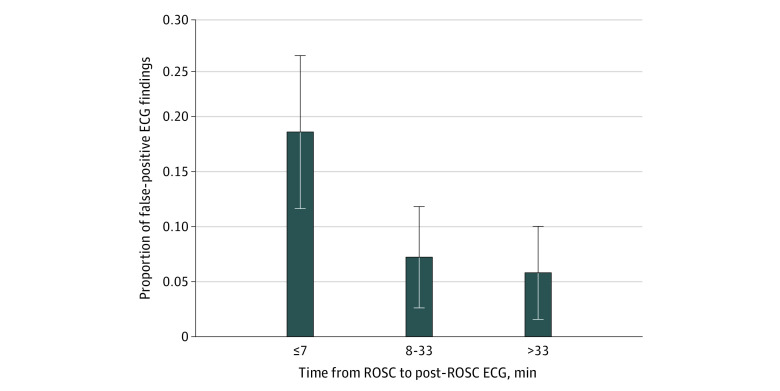
Percentage of False-Positive Electrocardiography (ECG) Findings for ST-Segment Elevation Myocardial Infarction (STEMI) in the Population in Whom a Percutaneous Coronary Angioplasty (PTCA) Was Not Performed The population was divided into 3 tertiles of time from return of spontaneous circulation (ROSC) to post-ROSC ECG acquisition (≤7 min, 8-33 min, and >33 min). In the logistic regression model, *P* < .001. Error bars indicate 95% CIs.

In analyses of the 3 tertiles of the time from ROSC to post-ROSC ECG acquisition (tertile 1: ≤7 minutes; tertile 2: 8-33 minutes; and tertile 3: >33 minutes), the percentage of false-positive ECG findings in the first tertile (18.5%) was significantly higher than that in the second tertile (7.2%; odds ratio [OR], 0.34; 95% CI, 0.13-0.87; *P* = .02) and third tertile (5.8%; OR, 0.27; 95% CI, 0.15-0.47; *P* < .001) (Figure 2; eFigure 1 in the Supplement gives time on a continuous scale). These differences remained significant when adjusting for sex (≤7 minutes: reference; 8-33 minutes: OR, 0.32; 95% CI, 0.12-0.85; *P* = .02; >33 minutes: OR, 0.26; 95% CI, 0.14-0.47; *P* < .001), age (≤7 minutes: reference; 8-33 minutes: OR, 0.34; 95% CI, 0.13-0.89; *P* = .03; >33 minutes: OR, 0.27; 95% CI, 0.15-0.46; *P* < .001), number of segments with ST-elevation (≤7 minutes: reference; 8-33 minutes: OR, 0.35; 95% CI, 0.15-0.81; *P* = .01; >33 minutes: OR, 0.28; 95% CI, 0.15-0.52; *P* < .001), QRS duration (≤7 minutes: reference; 8-33 minutes: OR, 0.35; 95% CI, 0.14-0.87; *P* = .02; >33 minutes: OR, 0.27; 95% CI, 0.15-0.48; *P* < .001), heart rate (≤7 minutes: reference; 8-33 minutes: OR, 0.35; 95% CI, 0.13-0.93; *P* = .04; >33 minutes: OR, 0.29; 95% CI, 0.15-0.55; *P* < .001), epinephrine administered (≤7 minutes: reference; 8-33 minutes: OR, 0.35; 95% CI, 0.13-0.98; *P* = .045; >33 minutes: OR, 0.27; 95% CI, 0.16-0.48; *P* < .001), shockable initial rhythm (≤7 minutes: reference; 8-33 minutes: OR, 0.35; 95% CI, 0.13-0.96; *P* = .04; >33 minutes: OR, 0.26; 95% CI, 0.15-0.46; *P* < .001), and 3 or more shocks administered (≤7 minutes: reference; 8-33 minutes: OR, 0.36; 95% CI, 0.13-1.00; *P* = .05; >33 minutes: OR, 0.27; 95% CI, 0.16-0.48; *P* < .001) in bivariable analyses (Table 3).

**Table 3.** Univariable Logistic Analysis for False-Positive Electrocardiography Findings According to Timing of Return of Spontaneous Circulation After Electrocardiography and Bivariable Analyses With Adjustments

Variable	OR (95%CI)	*P* value
**Overall**		
ROSC to ECG time, min		.04^a^
≤7	1 [Reference]	NA
8-33	0.34 (0.13-0.87)	.03
>33	0.27 (0.15-0.47)	<.001
**By Sex**		
ROSC to ECG time, min		<.001^a^
≤7	1 [Reference]	NA
8-33	0.32 (0.12-0.85)	.02
>33	0.26 (0.14-0.47)	<.001
Sex		
Female	1 [Reference]	NA
Male	1.92 (0.57-6.47)	.29
**By Age**		
ROSC to ECG time, min		<.001^a^
≤7	1 [Reference]	NA
8-33	0.34 (0.13-0.89)	.03
>33	0.27 (0.15-0.46)	<.001
Age, y		
≤62	1 [Reference]	NA
>62	0.88 (0.63-1.21)	.42
**By No. of segments with ST elevation**		
ROSC to ECG time, min		<.001^a^
≤7	1 [Reference]	NA
8-33	0.35 (0.15-0.81)	.01
>33	0.28 (0.15-0.52)	<.001
No. of segments with ST elevation		
≤1	1 [Reference]	NA
>1	1.25 (0.55-2.85)	.60
**By QRS duration**		
ROSC to ECG time, min		<.001^a^
≤7	1 [Reference]	NA
8-33	0.35 (0.14-0.87)	.02
>33	0.27 (0.15-0.48)	<.001
QRS duration, ms		
≤120	1 [Reference]	NA
>120	1.47 (1.02-2.12)	.04
**By heart rate**		
ROSC to ECG time, min		<.001^a^
≤7	1 [Reference]	NA
8-33	0.35 (0.13-0.93)	.04
>33	0.29 (0.15-0.55)	<.001
Heart rate, bpm		
≤100	1 [Reference]	NA
>100	1.74 (1.05-2.89)	.03
**By epinephrine dose administered**		
ROSC to ECG time, min		<.001^a^
≤7	1 [Reference]	NA
8-33	0.35 (0.13-0.98)	.045
>33	0.27 (0.16-0.48)	<.001
Epinephrine dose administered, mg		
≤1	1 [Reference]	NA
>1	1.22 (0.68-2.19)	.51
**By initial rhythm**		
ROSC to ECG time, min		<.001^a^
≤7	1 [Reference]	NA
8-33	0.35 (0.13-0.96)	.04
>33	0.26 (0.15-0.46)	<.001
Initial rhythm		
Not shockable	1 [Reference]	NA
Shockable	0.57 (0.13-2.5)	.45
**By No. of shocks administered**		
ROSC to ECG time, min		<.001^a^
≤7	1 [Reference]	NA
8-33	0.36 (0.13-1.00)	.05
>33	0.27 (0.16-0.48)	<.001
No. of shocks administered		
<3	1 [Reference]	NA
≥3	1.95 (1.27-2.98)	.002

Abbreviations: bpm, beats per minute; ECG, electrocardiography; NA, not applicable; OR, odds ratio; ROSC, return of spontaneous circulation.

^a^ *P* value of the difference among the 3 tertiles.

### Diagnostic Ability of Post-ROSC ECG by Time From ROSC

The positive predictive value of the ECG meeting STEMI criteria in predicting the need for PTCA intervention increased from 75.3% in the first tertile to 85.5% in the second tertile and 83.7% in the third tertile. Moreover, the specificity increased from 41% in the first tertile to 75% in the second tertile and 81.6% in the third tertile (eAppendix and eFigure 2 in the Supplement).

## Discussion

To our knowledge, this study was the first to investigate whether the timing of acquisition of post-ROSC ECG after OHCA was associated with the percentage of patients with post-ROSC ECG findings that met STEMI criteria but without obstructive coronary artery disease that was worthy of PTCA on angiography (false-positive ECG findings). The rate of false-positive ECG findings was significantly higher in the early phase after ROSC than in the later phase. This finding supports our hypothesis that, in the early post-ROSC phase, ECG findings could reflect not only the ischemia due to a coronary obstruction but also ischemia due to no blood flow and/or low blood flow during cardiac arrest.

Previous studies have suggested that a coronary lesion that is worthy of percutaneous revascularization is present in 40% to 60% of the patients resuscitated from an OHCA in whom the post-ROSC ECG did not show electrocardiographic signs of STEMI.^16,17,18^ Our findings support these data because 57.0% of the patients with a post-ROSC ECG that was not diagnostic of STEMI were treated with PTCA.

Although the literature provides evidence of a benefit of an immediate coronary angiography for patients with a post-ROSC ECG that is diagnostic of STEMI,^19^ a benefit of an immediate invasive approach has not yet been proven for patients with a post-ROSC ECG that is not diagnostic of STEMI except in the case of electrical or hemodynamic instability.

Previous retrospective studies suggested a benefit of immediate coronary angiography in patients with a post-ROSC ECG that is not diagnostic of STEMI.^17,20,21^ However, a recent randomized clinical trial reported that survival was similar when comparing an invasive with a delayed approach.^22^ This result could be explained by the balancing between a positive effect for those patients with a significant coronary stenosis even if they presented an ECG that was not diagnostic of STEMI and a potentially harmful effect for patients with a post-ROSC ECG that was not diagnostic of STEMI who were resuscitated from a cardiac arrest not attributable to cardiac ischemia, as in the case of brain hemorrhage or aortic dissection.

The evidence suggests that an urgent coronary angiography is associated with improved survival in the case of STEMI. On the other hand, the need for urgent coronary angiography is controversial in the absence of STEMI because the survival is similar regardless of an invasive or delayed approach.^23,24^

In light of these results, we believe that the precise diagnosis of STEMI is important for the correct management of patients who are resuscitated from OHCA and for the precise identification of patients for whom an urgent coronary angiography could be beneficial. However, the European Resuscitation Council, American Heart Association, and European Society of Cardiology guidelines, although stressing the key role of the ECG acquisition after ROSC, do not establish the timing for this.^6,8,9^

Our study showed that the timing of ECG recording was associated with minimizing the rate of false-positive ECG findings and therefore with correctly identifying patients who should undergo coronary angiography immediately. The percentage of false-positive ECG findings was 3 times greater when the ECG was acquired in the first 7 minutes after ROSC rather than after the eighth minute. The positive predictive value of ECG meeting STEMI criteria for predicting the need for PTCA increased from the first tertile of the ROSC to ECG time to the second and the third tertiles. Moreover, the rate of true-negative ECG findings (ie, findings that did not meet STEMI in patients who did not need PTCA) increased markedly between the first and second tertiles. This result can be reasonably explained considering that the absence of coronary flow during cardiac arrest induces ischemia, leading to an alteration of the ECG findings.^25,26,27^ If the post-ROSC ECG is performed too early, it could be reasonably affected by the deep ischemia induced by the period of no flow and/or low flow, resulting in a transmural myocardial ischemia that is not necessarily of coronary origin. To our knowledge, none of the studies on the immediate or delayed performance of coronary angiography after ROSC has considered the timing of ECG acquisition, which given the results of the present study, may have influenced the results of those studies.^16,17,18,19,20,21,22,23,24^

A percentage of patients with false-positive ECG findings (ie, patients who had ECG findings that met STEMI criteria but who did not require PTCA) could have experienced a myocardial infarction with nonobstructive coronary arteries. However, given that this is diagnosed in a small percentage of patients with acute myocardial infarction (approximately 6%)^28^ and that such cases would have been evenly distributed in the 3 tertiles of the ROSC to ECG time, it is reasonable to assume that myocardial infarction with nonobstructive coronary arteries did not affect our results.

In patients with false-negative ECG findings (ie, patients with a post-ROSC ECG that did not meet STEMI criteria but whose angiography showed obstructive coronary artery disease that was worthy of PTCA), the timing of an invasive approach was crucial to avoid delay in coronary lesion treatment. As recommended by the 2015 European Society of Cardiology guidelines for the management of patients with ventricular arrhythmias and the prevention of sudden cardiac death,^29^ examination in the intensive care unit should be considered to exclude noncoronary causes in patients with ECG findings that did not meet STEMI criteria, with a coronary angiography performed in the absence of an obvious noncoronary cause. This approach may allow for the avoidance of the potentially harmful effect of anticoagulation during coronary angiography in case of a noncardiac cause of the OHCA, such as brain hemorrhage or aortic dissection. Our results—suggesting to waiting 8 minutes after ROSC to acquire the ECG—are not against this type of approach, allowing in fact the reduction of the percentage of patients with false-positive ECG findings who, in the case of noncardiac causes of OHCA, could experience complications or death after an immediate invasive approach. Moreover, as suggested by our results, waiting 8 minutes may be associated with an increased rate of true-negative ECG findings. However, future studies may be beneficial to better comprehend which factors are associated with false-positive and false-negative ECG findings.

This study may have important clinical and scientific implications. From the clinical point of view, the results suggest waiting at least 8 minutes after ROSC for ECG acquisition or repeating the ECG after a few minutes in the case of an early ECG that is diagnostic of STEMI. Given that the median time between ROSC and coronary angiography reported in the literature ranged from 70 to 120 minutes,^16,19,22^ a similar finding in this study population, the repetition of the ECG after a few minutes did not affect this timing. Moreover, the acquisition of an eventual second ECG can be scheduled according to the different clinical and organizing settings, including just after loading in the ambulance but before leaving for the hospital or to reduce the prehospital time, such as in patients who are candidates for mechanical circulatory support, just after arriving at the hospital but before starting coronary angiography and/or mechanical support.

From the scientific point of view, the results of the PEACE study suggest that correction of the results of previous studies for the ROSC to ECG time may be needed to decrease the rate of false-positive ECG findings in the analyses. Moreover, prospective, systematic, and larger studies that also acquire serial ECGs in the same patients appear to be needed to confirm and clarify our findings by focusing on early ECG findings and whether it is possible to identify electrocardiographic markers for a better discrimination of true-positive ECG findings from false-positive ECG findings for STEMI.

### Strengths and Limitations

This study has strengths. The multicenter design involved hospitals in different geographical areas and thus decreased the risk of bias typical of monocentric or multicenter studies in areas with similar prehospital and hospital settings. Moreover, data on the prehospital treatment of the patients included in the study were also available, showing that in this study population, most OHCAs occurred in a public place and were witnessed with a shockable presenting rhythm. This finding was expected because these factors are known to be associated with OHCA survival,^30,31^ and the study population comprised patients who survived to hospital arrival.

This study also has limitations. First, it was a retrospective study, and the sample size was limited. Second, the end point at the coronary angiography was the execution of a percutaneous angioplasty and not the identification of a culprit lesion (acute coronary occlusion or unstable lesion). This choice was derived from the inhomogeneity of the criteria for defining culprit lesions across various centers, especially considering the clinical context of urgency in which angiography is performed that often does not allow the application of techniques such as intravascular ultrasonography and optical coherence tomography. Because of the retrospective nature of this study, it was not possible to obtain these criteria in a homogeneous way from the participating centers. However, given the STEMI guidelines^9^ and the median time from ECG acquisition to coronary angiography, it seems reasonable to assume that the interventional cardiologist treated the lesion or lesions that were considered the culprit. Third, the diagnosis of STEMI was based on only the ECG, and no advanced imaging technique (ie, cardiac magnetic resonance imaging) was used to confirm or exclude nonobstructive disease such as myocardial infarction with nonobstructive coronary arteries. Fourth, a post-ROSC ECG was not available in 25% of the patients because of several factors, including the ECG not being performed before arrival in the catheterization laboratory, the ECG not being interpretable for artifacts, the ECG diagnostic of STEMI being performed before OHCA in the case of EMS-witnessed OHCA, or the ECG not being retrievable in the patient's medical records. These factors represent the typical limitations of a retrospective study, may have affected the results, and may represent a stimulus to carry out a prospective study about this topic to overcome these limitations. Moreover, a coronary angiography was not performed during the hospital stay in a substantial percentage of patients given the number of patients who died between hospital arrival and performance of coronary angiography. Fifth, the quality of resuscitation was not evaluated, which could represent a possible confounder because it is associated with the propensity for visceral ischemia and, in particular, cardiac ischemia.

## Conclusions

In this cohort study, the early acquisition of the post-ROSC ECG in patients who were resuscitated from an OHCA was associated with a higher rate of patients with post-ROSC ECG findings that met STEMI criteria but who did not have obstructive coronary artery disease worthy of PTCA on angiography. Therefore, delaying the post-ROSC ECG by at least 8 minutes after ROSC or repeating the acquisition if the first ECG was diagnostic of STEMI and was acquired early after ROSC may be reasonable to correctly identify patients who may benefit from an immediate rather than a delayed coronary angiography.

## Notes

Supplement. **eTable 1.** Comparison of Patients’ and OHCAs’ Characteristic According to the Tertiles of ROSC-ECG Time **eTable 2.** ECG and Coronary Angiography Characteristics According to the Tertiles of ROSC-ECG Time **eFigure 1.** Shape of Risk of False Positive ECGs for STEMI Rate Over Time (Fractional Polynomials) **eFigure 2.** Positive Predictive Value and Specificity of the ECG Meeting STEMI Criteria in Predicting the Need of PTCA Intervention According to the Three Tertiles of the Time From ROSC to Post-ROSC ECG **eAppendix.** Criteria for Coronary Angiography in Post-ROSC Patients
