# Supplementary material for: Association of Timing of Electrocardiogram Acquisition After Return of Spontaneous Circulation With Coronary Angiography Findings in Patients With Out-of-Hospital Cardiac Arrest
Source: JAMA Netw Open. 2021 Jan 11;4(1):e2032875. doi: 10.1001/jamanetworkopen.2020.32875 (PMC7801935; doi:10.1001/jamanetworkopen.2020.32875)
Supplement: Supplement. — eTable 1. Comparison of Patients’ and OHCAs’ Characteristic According to the Tertiles of ROSC-ECG Time eTable 2. ECG and Coronary Angiography Characteristics According to the Tertiles of ROSC-ECG Time eFigure 1. Shape of Risk of False Positive ECGs for STEMI Rate Over Time (Fractional Polynomials) eFigure 2. Positive Predictive Value and Specificity of the ECG Meeting STEMI Criteria in Predicting the Need of PTCA Intervention According to the Three Tertiles of the Time From ROSC to Post-ROSC ECG eAppendix. Criteria for Coronary Angiography in Post-ROSC Patients [file jamanetwopen-e2032875-s001.pdf]

Supplementary Online Content

Baldi E, Schnaubelt S, Caputo ML, et al. Association of timing of electrocardiogram acquisition after return of spontaneous circulation with coronary angiography findings in patients with out-of-hospital cardiac arrest. *JAMA Netw Open*. 2021;4(1):e2032875. doi:10.1001/jamanetworkopen.2020.32875

eTable 1. Comparison of Patients' and OHCAs' Characteristic According to the Tertiles of ROSC-ECG Time

eTable 2. ECG and Coronary Angiography Characteristics According to the Tertiles of ROSC-ECG Time

eFigure 1. Shape of Risk of False Positive ECGs for STEMI Rate Over Time (Fractional Polynomials)

eFigure 2. Positive Predictive Value and Specificity of the ECG Meeting STEMI Criteria in Predicting the Need of PTCA Intervention According to the Three Tertiles of the Time From ROSC to Post-ROSC ECG

eAppendix. Criteria for Coronary Angiography in Post-ROSC Patients

This supplementary material has been provided by the authors to give readers additional information about their work.

©2021 Baldi E et al. *JAMA Network Open*

eTable 1. Comparison of Patients' and OHCAs' Characteristic According to the Tertiles of ROSC-ECG Time

Variable	ROSC-ECG time ≤7 mins (n=123)	ROSC-ECG time 8-33 mins (n=126)	ROSC-ECG time >33 mins (n=121)	p-value
Center, n (%)				<.001
Pavia	66 (53.7)	49 (38.9)	6 (5)	
Lugano	21 (17.1)	8 (6.3)	9 (7.4)	
Vienna	36 (29.3)	69 (54.8)	106 (87.6)	
Males, n (%)	89 (72.4)	104 (82.5)	94 (77.7)	.16
Age, years median [IQR]	63 [52-70]	61 [54-70]	61 [52-70]	.76
OHCA location, n (%)				.28
Home	75 (61)	61 (48.4)	51 (42.1)	
Public buildings	22 (17.9)	30 (23.8)	36 (29.8)	
Work/Office	1 (0.8)	3 (2.4)	5 (4.1)	
Street	18 (14.6)	22 (17.5)	20 (16.5)	
Sport	1 (0.8)	2 (1.6)	0 (0)	
Others	4 (3.3)	5 (4)	7 (5.8)	
Unknown	2 (1.6)	3 (2-4)	2 (1.7)	
EMS arrival time, mins median [IQR]	9 [7-12]	9 [7-12]	8.5 [7-11]	.30
OHCA witnessed, n (%)				.46
No	17 (13.8)	16 (12.7)	20 (16.5)	
Yes, by bystander	88 (71.5)	89 (70.6)	90 (74.4)	
Yes, by EMS	18 (14.6)	21 (16.7)	11 (9.1)	
Bystander CPR †, n (%)	68 (66)	79 (75.2)	86 (78.2)	.11
First shockable rhythm, n (%)	103 (83.7)	111 (88.8)	98 (81.7)	.19

Epinephrine administered, mg median [IQR]	2 [0-3]	0 [0-2]	1 [0-3]	.01
Number of shocks administered, median [IQR]	3 [1-5]	2 [1-4]	2 [1-4]	.47
Survival at hospital discharge, n (%)	77 (63.1)	97 (78.2)	70 (59.3)	.01
Survival at hospital discharge with good neurological outcome (CPC 1 or 2)	62 (80.5)	85 (88.5)	64 (91.4)	.04

† Excluding EMS-witnessed

©2021 Baldi E et al. *JAMA Network Open*

eTable 2. ECG and Coronary Angiography Characteristics According to the Tertiles of ROSC-ECG Time

Post-ROSC ECG items	ROSC-ECG time ≤7 mins (n=123)	ROSC-ECG time 8-33 mins (n=126)	ROSC-ECG time >33 mins (n=121)	p-value
ROSC-ECG time, mins [IQR]	4 [2-6]	15.9 [10-23.2]	48 [40-62.2]	<.001
Rhythm, n (%)				<.001
Sinus rhythm	71 (57.7)	95 (75.4)	103 (85.8)	
Atrial fibrillation / Atrial tachycardia	38 (30.9)	27 (21.4)	15 (12.5)	
Junctional or ventricular rhythm	13 (10.6)	3 (2.4)	0 (0)	
Paced	1 (0.8)	1 (0.8)	2 (1.7)	
ECG diagnostic for STEMI, n (%)	92 (74.8)	63 (50)	43 (35.5)	<.001
Heart rate, bpm [IQR]	101 [84-130]	99.5 [79-116]	91 [74.7-106]	<.001
QRS duration, msec [IQR]	120 [100-140]	110 [90-132]	114 [96-144]	.42
QTc value, msec [IQR]	450 [413-490]	452 [423-485]	484 [456-515]	<.001
Intraventricular conduction, n (%)				<.01
Normal	75 (61)	88 (69.8)	77 (63.6)	
Left bundle branch block	12 (9.8)	12 (9.5)	21 (17.4)	
Right bundle branch block	24 (19.5)	18 (14.3)	22 (18.2)	
Bifascicular block	8 (6.5)	0 (0)	0 (0)	
Others	4 (3.3)	8 (6.3)	1 (0.8)	
Arrhythmias, n (%) †				<.01
None	60 (84.5)	85 (90.4)	103 (100)	
Ventricular ectopy	9 (12.7)	5 (5.3)	0 (0)	
Supraventricular ectopy	2 (2.8)	4 (4.3)	0 (0)	

©2021 Baldi E et al. *JAMA Network Open*

Numbers of segments with ST elevation, n (%)				<.001
None	26 (21.1)	56 (44.4)	65 (53.7)	
One	43 (35)	33 (26.2)	28 (23.1)	
Two	37 (30.1)	33 (26.2)	14 (11.6)	
Three	16 (13)	2 (1.6)	10 (8.3)	
Four	1 (0.8)	2 (1.6)	4 (3.3)	
**Coronary angiography items**				
ECG-angiography time, mins [IQR]	90 [64-131]	104.5 [65-264]	107 [64-403.5]	.17
Normal coronary angiography, n (%)	18 (14.6)	22 (17.5)	31 (25.6)	.08
Number of vessels with significant stenosis, n (%)				.47
None	21 (17.1)	27 (21.4)	32 (26.4)	
One-vessel	38 (30.9)	39 (31)	36 (29.8)	
Two-vessels	30 (24.4)	31 (24.6)	20 (16.5)	
Three-vessels	34 (27.6)	29 (23)	33 (27.3)	
Number of vessels treated with PTCA, n (%)				.21
None	39 (31.7)	36 (28.6)	38 (31.4)	
One-vessel	58 (47.2)	55 (43.7)	40 (33.1)	
Two-vessels	14 (11.4)	18 (14.3)	20 (16.5)	
Three-vessel	12 (9.8)	17 (13.5)	23 (19)	

† Considering only ECG with sinus rhythm

©2021 Baldi E et al. *JAMA Network Open*

eFigure 1. Shape of Risk of False Positive ECGs for STEMI Rate Over Time (Fractional Polynomials)

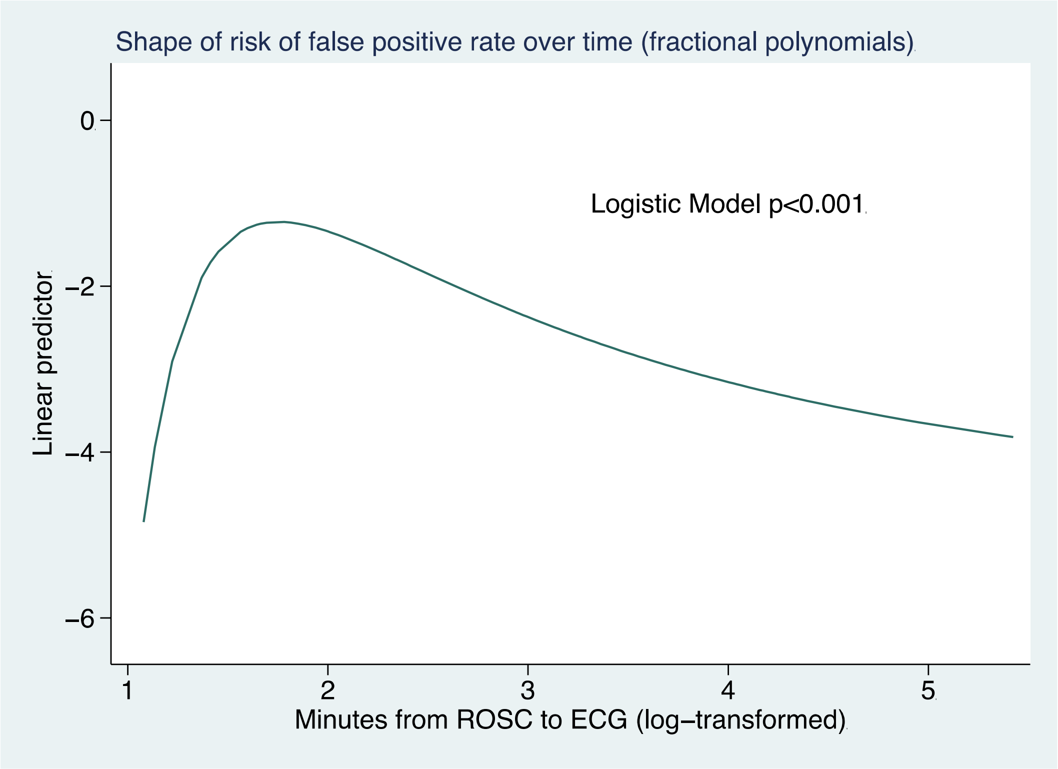

©2021 Baldi E et al. *JAMA Network Open*

eFigure 2. Positive Predictive Value and Specificity of the ECG Meeting STEMI Criteria in Predicting the Need of PTCA Intervention According to the Three Tertiles of the Time From ROSC to Post-ROSC ECG

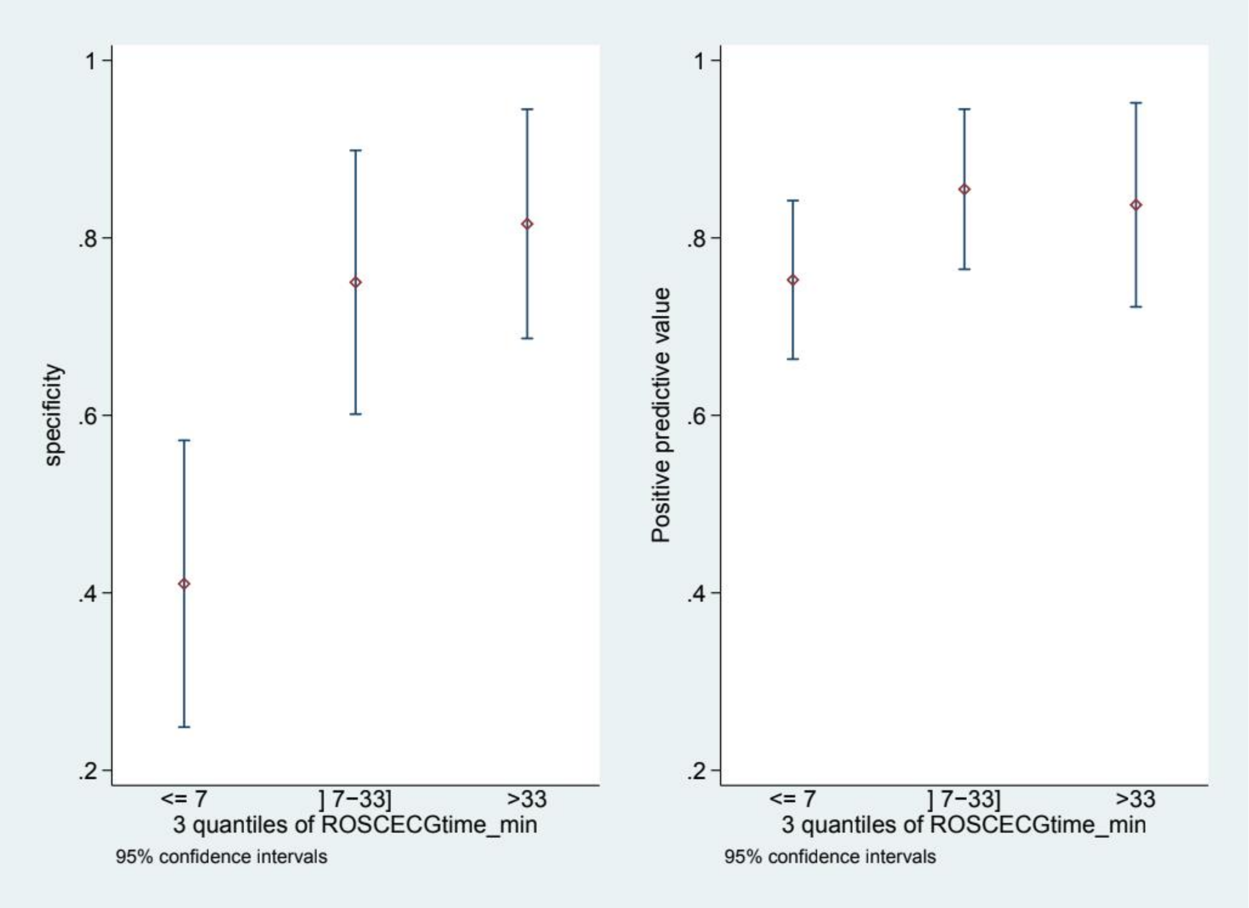

©2021 Baldi E et al. *JAMA Network Open*

eAppendix. Criteria for Coronary Angiography in Post-ROSC Patients

Fondazione IRCCS Policlinico San Matteo, Pavia, Italy: A primary percutaneous coronary intervention (PCI) is performed in patients resuscitated from out-of-hospital cardiac arrest (OHCA) and with a post-ROSC ECG meeting STEMI criteria. Regarding patients with a post-ROSC ECG not meeting STEMI criteria, an urgent angiography is performed only in those considered at high suspicion of ongoing myocardial ischemia by the cardiologist, which usually takes into account the age and the clinical history (including symptoms before cardiac arrest).

Cardiocentro Ticino, Lugano, Switzerland: A primary percutaneous coronary intervention (PCI) is performed in patients resuscitated from out-of-hospital cardiac arrest (OHCA) with a first shockable rhythm. Regarding other patients, the decision to perform an urgent angiography is done by the cardiologist taking into account, beside the ECG, the age and the clinical history (including symptoms before cardiac arrest).

Medical University of Vienna, Vienna, Austria: A primary percutaneous coronary intervention (PCI) in out-of-hospital cardiac arrest (OHCA) patients is performed whenever patients either meet ST-elevation myocardial infarction (STEMI) criteria in an ECG post-ROSC or when patients do not meet STEMI-criteria, but are highly suspicious of acute coronary syndrome or cardiac etiology of OHCA. This latter decision is made in a multi-professional team consisting of emergency medicine physicians and cardiologists. Multiple criteria such as symptoms prior to OHCA, co-morbidities, age etc. are taken into account for the decision.

©2021 Baldi E et al. *JAMA Network Open*
